# Ganoderic Acid A targeting *leucine‐rich repeat kinase 2* involved in Parkinson's disease–A computational study

**DOI:** 10.1002/agm2.12235

**Published:** 2022-12-20

**Authors:** Faizan Ahmad

**Affiliations:** ^1^ Department of Medical Elementology and Toxicology Jamia Hamdard University Delhi India

**Keywords:** *Ganoderma lucidium*, molecular dynamics simulation, MMGBSA, Parkinson's disease

## Abstract

**Objective:**

This study aims to find the most promising *Ganoderma lucidium* targeting LRRK2 involved in PD.

**Methods:**

First ADMET analysis was performed for five compounds followed by molecular docking of each compound. Then, we perform molecular dynamics simulation of all five compounds and finally MMGBSA of all five compounds.

**Results:**

Based on molecular dynamics and MMGBSA result we reach the conclusion that Ganoderic Acid A (GAA) is the most promising compound targeting LRRK2. Therefore, GAA needs further validation through in vitro and in vivo studies.

**Conclusion:**

*Ganoderma lucidum* exhibits cytotoxic, hepatoprotective, antioxidative, anticancer, and antinociceptive activities. This study predicted that *Ganoderma lucidum* could even be used to treat neurological disorders like PD. This study suggest that the best‐identified molecule against LRRK2 is GAA and it needs rigorous in vitro and in vivo validations.

## Graphical abstract

Parkinson's Disease is the second most neurological disorder Resihi mushroom has multiple medicinal properties LRRK2 is the one of the most emerging therapeutic target Molecular dynamics and MMGBSA results conclude that Ganoderic Acid A is the most promising compound targeting LRRK2.

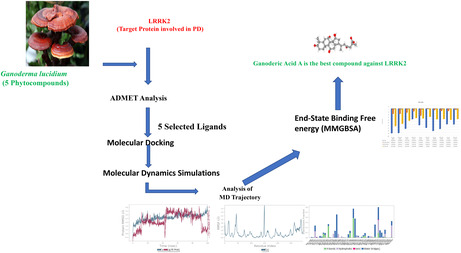

## 1 INTRODUCTION

Parkinson's disease (PD) is the second most prevalent cause of movement abnormalities in the elder people after Alzheimer's disease (AD). Dopamine levels in the brain decrease as substantia nigra cells die, which is detrimental to movement control centres. As the symptoms of PD worsen, it starts affecting one side of the body first and then the other, resulting in shaking and weakness of the lips, hands, arms, legs, and trunk, as well as slow movement and poor body control and coordination. In extreme situations, patients may experience difficulties walking and chewing, as well as depression and sleep disorders. According to study, mutations in the genes *SNCA*, *PARK2*, *PINK1*, *DJ1*, *LRRK2*, and *ATP13A2* disrupt dopaminergic pathway production and reception, resulting in PD. There are multiple targets in drug discovery models for PD. Predictions indicate that drugs designed for specific target populations will have a 50% greater failure rate as well as provide 50% less value than treatments developed for established target populations. As a result, much of the drug research in PD has focused on increasing the effectiveness of Levodopa (L‐dopa) while decreasing its side effects. However, in the case of PD, L‐dopa merely gives symptomatic relief and has no effect on the neuronal multisystem dysfunction and degeneration that occurs in the condition.^1^, ^2^, ^3^, ^4^, ^5^, ^6^, ^7^ LRRK2 was first reported in 2004 in Japan that pathogenic variants of the LRRK2 gene cause autosomal dominant PD. LRRK2 mutations cause cellular malfunction and, eventually, neurodegeneration will lead to the development of new pharmaceutical therapies and preclinical studies indicate that reduction of LRRK2 activity or expression is neuroprotective; small‐molecule LRRK2 inhibitors and antisense oligonucleotides have been developed and are now considered suitable for clinical exposure.^8^ In this computational study, we have considered *Ganoderma lucidum*, also known as “Reishi mushroom,” under the category of medicinal mushroom. *Ganoderma lucidum* has immune‐stimulating activities, antiinflammatory and antiallergenic properties for cancer, and can be used for treating and managing neurological disorders like PD and AD. In this study, we took the five most promising molecules in the Reishi mushroom extract: Ganoderic Acid A, Ganoderic Acid D, Ganoderic Acid F, Ganoderenic Acid B, and Ganoderenic Acid D.^9^, ^10^, ^11^ In our study, we performed molecular docking for the five compounds mentioned above, and finally, we performed molecular dynamics simulation for all the five compounds. Ultimately, this study aims to find the most promising compound from *Ganoderma lucidum* extract, which can be used for in vitro and in vivo studies.

## 2 METHODS

### 2.1 ADMET analysis

To calculate the pharmacokinetic and pharmacological properties of these five compounds, SWISSADME was used.

### 2.2 Molecular docking

Docking Studies were performed with AutoDock 4.2.6, using the standard procedures. The 3D coordinates of the LRRK2 protein were retrieved from the RCSB database with the PDB ID 7LHT and five ligands from the PubChem database. A grid box with x, y, and z directions was generated, keeping the grid spacing at 0.375 Å, and flexible multiple ligand docking was performed using a Perl script. The outputs were analyzed and visualized in Discovery Studio Visualizer.

### 2.3 Molecular dynamics simulation

Desmond, a program by Schrdinger LLC, was used to model molecular dynamics in 100 ns. Structural studies were performed on the protein–ligand complex as the first step in the molecular dynamics model preparation. Under static conditions, molecular connectivity studies can predict the binding state of ligands. The substrate attachment creates a static picture of the crucial position of an individual molecule in the active site of the protein. At the same time, the molecular dynamics simulation uses Newton's classical equation of motion to calculate the movement of atoms over time. The binding state of the ligands in the physiological environment was predicted using simulations. The accompanying protein–ligand complex using the Protein Preparation Wizard or Maestro software to optimize and minimize the structure of the protein–ligand complex. All systems were prepared using the System Builder tool. TIP3P (Transferable Intermolecular Interaction Potential 3 Points) was chosen as the orthorhombic solvent model because of its simplicity. Simulations were performed using the OPLS 2005 force field. 0.15 M sodium chloride (NaCl) was applied to simulate physiological conditions. The NPT ensemble was used throughout the simulation, with a temperature of 300 K and a pressure of 1 atm. Models were built more dynamically before simulation.^12^, ^13^, ^14^, ^15^, ^16^

### 2.4 Molecular mechanics generalized born and surface area (MMGBSA) calculations

The binding free energies (Gobind) of the anchored complexes were calculated using the first Molecular Mechanics Generalized Born Surface Area (MMGBSA) module (Schrodinger suite, LLC, New York). Binding free energies were calculated using OPLS 2005 force field, VSGB solvent model, and rotamer search methods18. After performing MD, a period of 10 ns was used to select the MD orbital frame. The total free binding energy is calculated using the below‐mentioned formula:

$$\text{∆Gobind}=\text{Gcomplex}–\left(G-\text{protein}+\text{Gligand}\right)$$

where ∆Gobind = binding free energy, Gcomplex = free energy of the complex, G‐protein = free energy of the target protein, and Gligand = free energy of the ligand.

## 3 RESULTS

### 3.1 ADMET analysis

The ADMET analysis of the selected molecules was done. ADME abbreviation stands for Absorption, Distribution, Drug Metabolism, and Excretion. It describes the absorption, distribution, drug metabolism, and excretion of a substance or medication. Solubility and permeability are critical characteristics that influence the oral absorption rate. As soon as the medication is dissolved, it circulates throughout the body and is divided into compartments. Drug distribution into tissues and organs is governed by pharmacokinetic characteristics such as plasma protein binding, lipophilicity, and phospholipids, three essential variables to consider. Drug metabolism is the process by which a drug molecule is broken down or altered enzymatically, followed by excretion. The most important metric for determining pharmaceutical excretion in humans is renal clearance, which is essential for assessing pharmaceutical excretion in humans. Lipinski's five rule also referred to as the Pfizer rule of five, requires that oral pharmaceuticals have a molecular weight of less than 500, fewer than five hydrogen bond donors and acceptors, and a distribution ratio of less than five to be evaluated for approval. All the five compounds follow Lipinski's rule of five. The result of the ADMET analysis of five compounds is mentioned in Table 1.

**TABLE 1:** Pharmacokinetic and ADMET analysis of five compounds

Parameters	Ganoderic Acid A	Ganoderic Acid D	Ganoderic Acid F	Ganoderenic Acid B	Ganoderenic Acid D
Molecular weight	516.67 g/mol	514.65 g/mol	570.67 g/mol	514.65 g/mol	512.63 g/mol
Number of H bond acceptor	7	7	9	7	7
Number of H bond donor	3	2	1	3	2
CYP1A2 inhibitor	No	No	No	No	Yes
CYP2C19 inhibitor	No	No	No	No	No
CYP2C9 inhibitor	No	No	No	No	No
CYP2D6 inhibitor	No	No	No	No	No
CYP3A4 inhibitor	Yes	No	No	Yes	No
Log Kp (Skin permeation)	−7.90 cm/s	−8.10 cm/s	−8.16 cm/s	−7.76 cm/s	−7.98 cm/s
Lipinski's rule of five	Yes	Yes	Yes	Yes	Yes

### 3.2 Molecular docking

**FIGURE 1:**
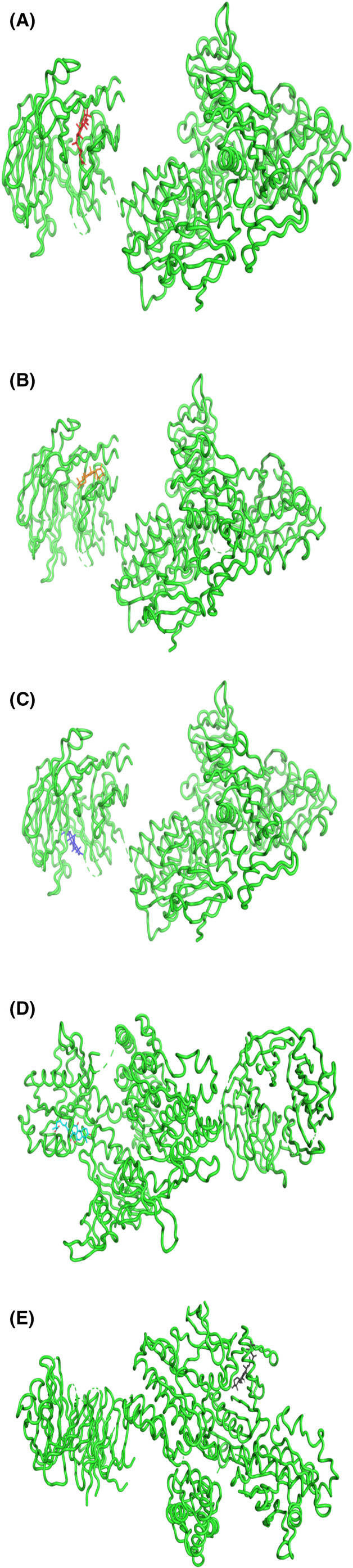
The 3D interaction diagram of the docked ligand with target protein LRRK2

The blind molecular docking of the selected five molecules from the *Ganoderma lucidum* was done on LRRK2 target protein. The binding affinity of the five compounds lies between −3.0 to −3.4 kcal/mol as mentioned in Table 2, and their 2D interactions with the target protein active site residues are shown in Figure 1. The Ganoderic Acid A (471002) is interacting at the residues of scaffold (ankyrin) domain of LRRK2 while Ganoderic Acid D (14109405), Ganoderic Acid F (23247895), and Ganoderenic Acid B (78074039) are interacting with the CoR domain of LRRK2 that is responsible for dimerization of the LRRK2 monomers whereas Ganoderenic acid D (76378890) is interacting with the active site residues of RoC GTPase domain. Based on their hydrophobic and hydrogen bond interactions as shown in Figure 1, these complexes were further subjected to molecular dynamics simulation.

**TABLE 2:** Docking score of five compounds of *Ganoderma lucidum* extract onto the target protein LRRK2

Compound Name	Pubchem ID	Binding affinity (kcal/mol)
Ganoderic Acid A	471,002	−3.0
Ganoderic Acid D	14,109,405	−3.2
Ganoderic Acid F	23,247,895	−3.3
Ganoderenic Acid B	78,074,039	−3.4
Ganoderenic Acid D	76,378,890	−.3.0

### 3.3 Molecular dynamics simulation

**FIGURE 2:**
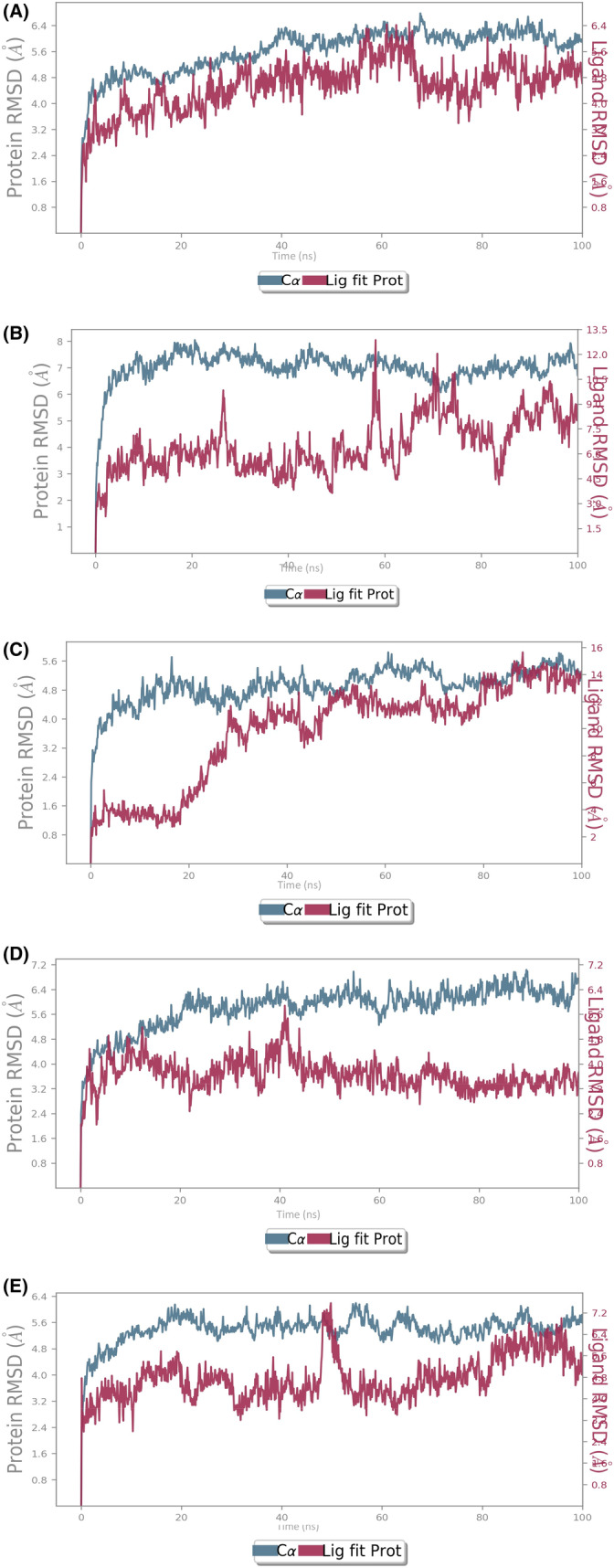
RMSD of (A) 471,002‐6XR4 (B) 14,109,405‐6XR4 (C) 23,247,895‐6XR4 (D) 78,074,039‐6XR4 (E) 76,378,890‐6XR4

The assessment of the complex stability between the LRRK2 and the screened molecules from the *Ganoderma lucidum* extract was done by subjecting them to molecular dynamics simulation in order to observe the dynamic interactions at atomic level. The RMSD of the LRRK2 (Figure 2) in these complexes ranged from 4.8 to 8.0 Å with the lowest RMSD observed in complex with Ganoderic Acid F (23247895) and the highest RMSD was observed in case of Ganoderic Acid D (14109405). For the screened ligands, their RMSD fitted onto the C‐alpha of LRRK2 ranged from 2 to 14 Å where Ganoderic Acid F (23247895) showed the highest fluctuations. Overall, the complexes reached to the equilibrium and stable state by the end of the simulation time interval.

### 3.4 Molecular mechanics generalized born and surface area (MMGBSA) calculations

The binding free energies (Gobind) of the anchored complexes were calculated using the first Molecular Mechanics Generalized Born Surface Area (MMGBSA) module (Schrodinger suite, LLC, New York). Binding free energies were calculated using OPLS 2005 force field, VSGB solvent model, and rotamer search methods18. After performing MD, a period of 10 ns was used to select the MD orbital frame. The total free binding energy is calculated using the below‐mentioned formula:

$$\text{∆Gobind}=\text{Gcomplex}–\left(G-\text{protein}+\text{Glgand}\right).$$

where ∆Gobind = binding free energy, Gcomplex = free energy of the complex, G‐protein = free energy of the target protein, and Gligand = free energy of the ligand.

**FIGURE 3:**
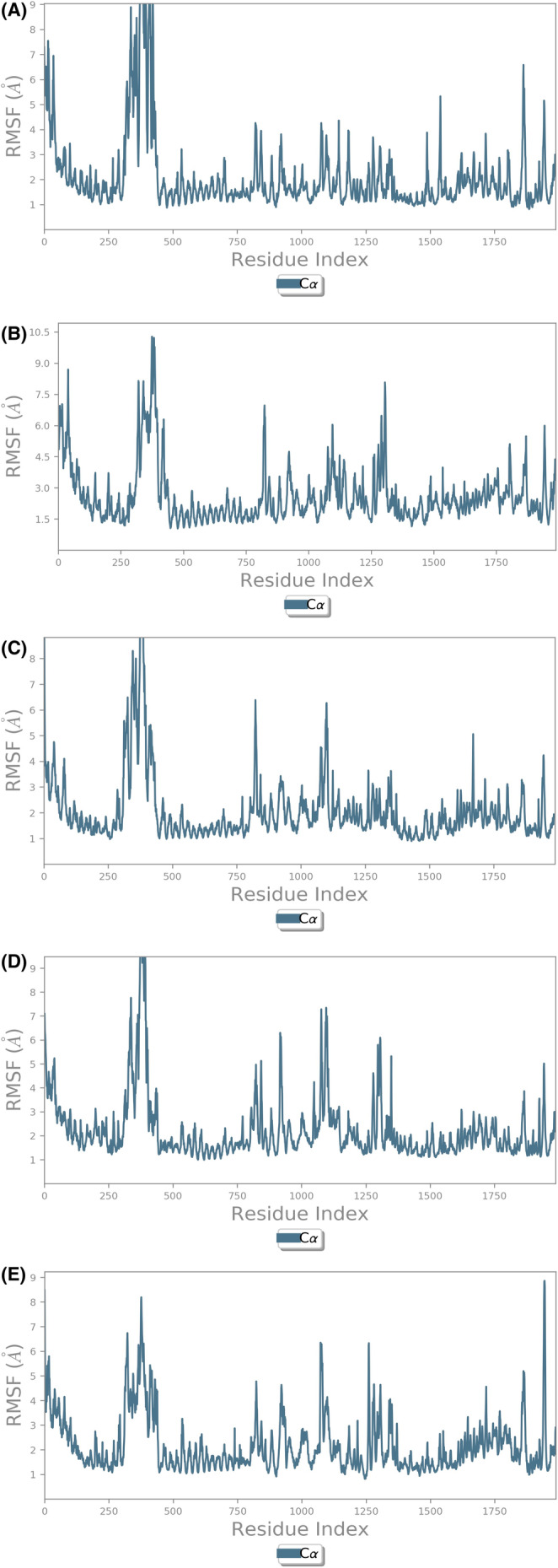
RMSF of (A) 471,002‐6XR4 (B) 14,109,405‐6XR4 (C) 23,247,895‐6XR4 (D) 78,074,039‐6XR4 (E) 76,378,890‐6XR4

**FIGURE 4:**
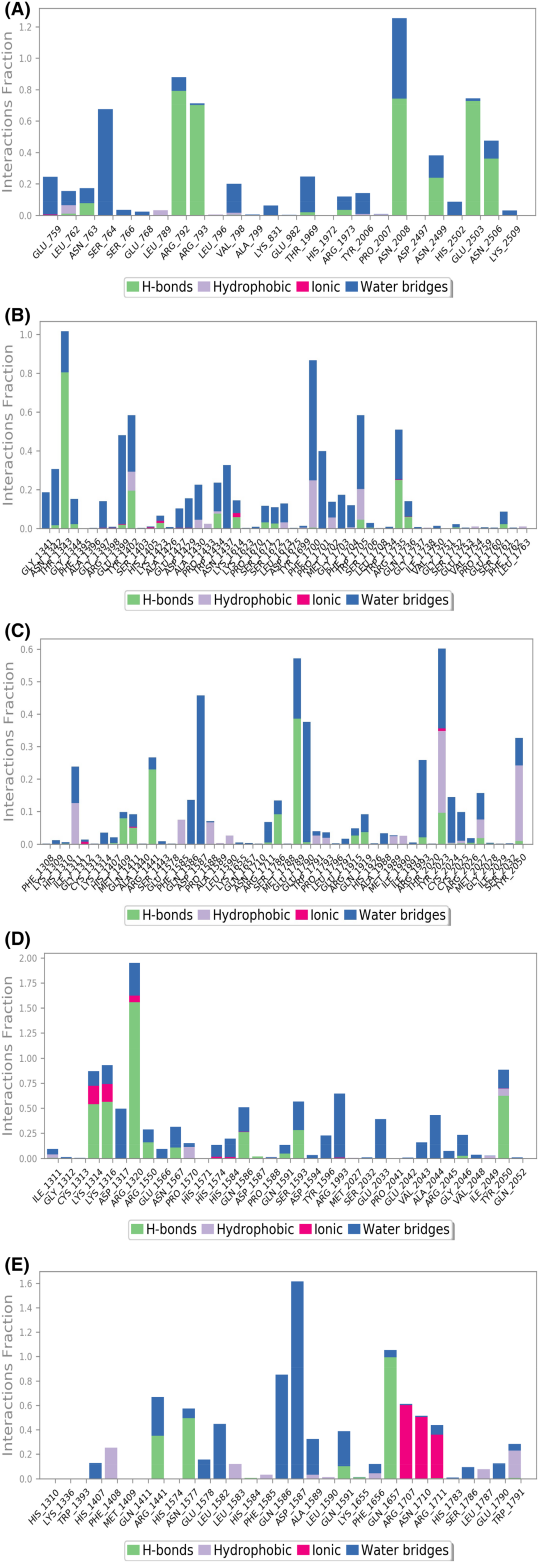
Histogram of (A) 471,002‐6XR4 (B) 14,109,405‐6XR4 (C) 23,247,895‐6XR4 (D) 78,074,039‐6XR4 (E) 76,378,890‐6XR4

**FIGURE 5:**
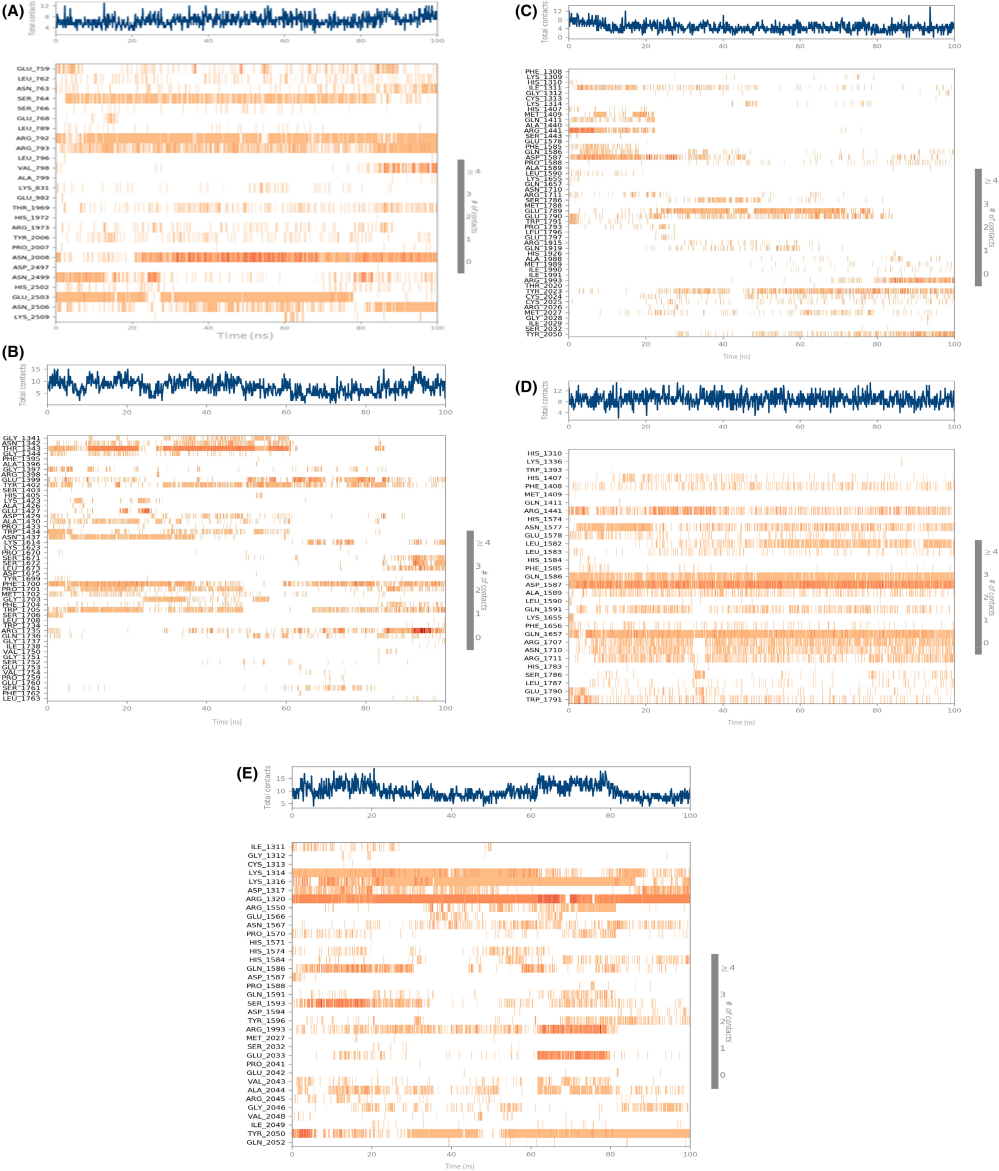
Contact Timeline of (A) 471,002‐6XR4 (B) 14,109,405‐6XR4 (C) 23,247,895‐6XR4 (D) 78,074,039‐6XR4 (E) 76,378,890‐6XR4

**FIGURE 6:**
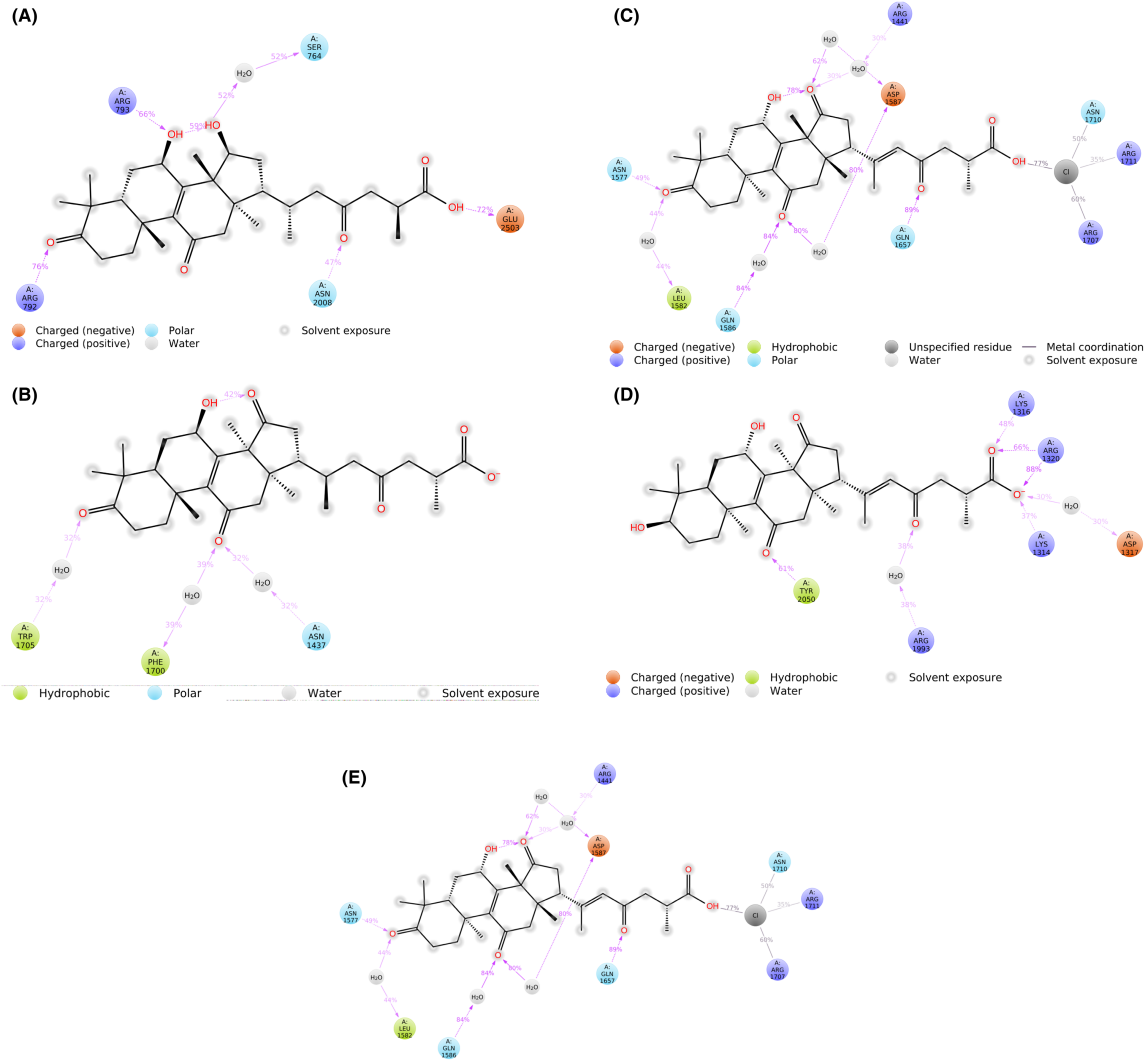
Ligand protein interactions of (A) 471,002‐6XR4 (B) 14,109,405‐6XR4 (C) 23,247,895‐6XR4 (D) 78,074,039‐6XR4 (E) 76,378,890‐6XR4

The RMSF is the measure of the fluctuations in the amino acid residues observed over the simulation time period. The RMSF of the amino acid residues of LRKK2 in complex with the ligand molecules (Figure 3) ranged from 1–9 Å where highest fluctuations were observed in the scaffold domain (Armadillo, ankyrin, and LRR) residues followed by RoC domain residues. The interaction histograms showed that the predominant interactions between the screened ligand molecules and LRRK2 domains were hydrogen bonds and salt bridge interactions followed by hydrophobic interactions while Ganoderenic Acid B (78074039) showed significant amount of ionic interactions at the CoR domain of LRRK2 (Figure 4). The protein–ligand contact timeline plots (Figure 5) showed that the average number of contacts between the LRRK2 and ligand molecules ranged from 4 to 12 contacts where minimum 60% occupancy of contacts was maintained with the critical residues in all the cases except 23,247,895 where the occupancy of contacts over the timeline was comparatively less. The contacts between the protein residue and ligands over the simulation time is shown in Figure 6.

**FIGURE 7:**
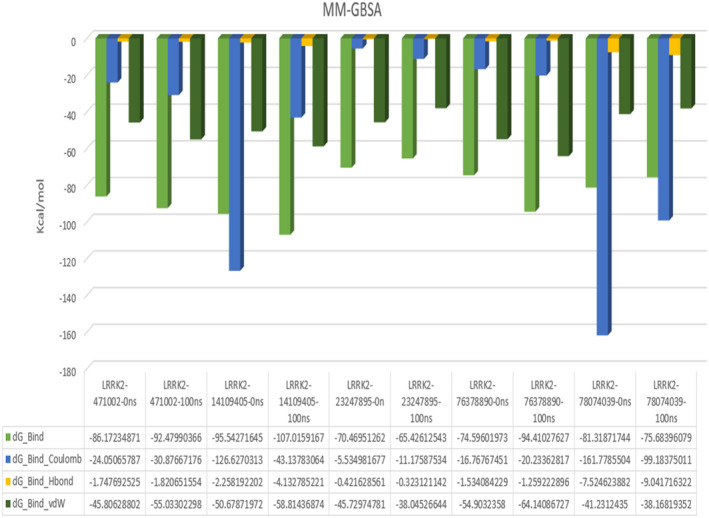
MMGBSA of (A) 471,002‐6XR4 (B) 14,109,405‐6XR4 (C) 23,247,895‐6XR4 (D) 78,074,039‐6XR4 (E) 76,378,890‐6XR4

The end‐state binding free energy calculations were performed for the initial and final pose of LRRK2 and screened ligands where the highest affinity was observed between LRRK2 and 14,109,405 followed by 76,378,890, 471,002, 78,074,039 and 723,247,895 as shown in Figure 7. Overall, all the ligands showed negative binding energy that makes their interactions with LRRK2 thermodynamically favorable.

The further validation of the protein–ligand binding affinity was done by calculating the MMGBSA binding free energies for the complexes (Figure 7). The best identified molecule is 471,002, showed the highest binding affinity after 100 ns with the energy scores of −92.47 kcal/mol, while the binding affinity of other ligand molecules were −65.42, −94.41, and −75.68 kcal/mol for 76,378,890, 1,410,905, and 23,247,895 respectively.

## 4 DISCUSSION

Molecular docking is the most extensively utilized technique since it allows tiny compounds to interact with targets at the atomic level. Using this technique, we can better understand small molecule behavior in specific protein targets and predict the structure of ligand–receptor complexes, which is critical for drug development. In this study, we took the five most potent molecules of *Ganoderma lucidum* extract, which include Ganoderic Acid A (GAA), Ganoderic Acid D, Ganoderic Acid F, Ganoderenic Acid D, and Ganoderenic Acid B, which shows the binding affinity of −3.0, −3.2, −3.3, −3.4, and −3.0 kcal/mol, respectively. We performed further studies like molecular dynamics simulation and MMGBSA; we concluded that GAA is the most promising Ganoderma lucidium extract. Ganoderma lucidum has medicinal properties and is even used for cancer, but in this study we are focused on PD. Recent study conducted by Ruiping et al. (2011) shows that *Ganoderma lucidium* protects dopaminergic neurons degeneration through inhibition of microglial activation.^17^ Study conducted by Sana et al. (2021) shows that *Ganoderma lucidium* has neuroprotective and anti‐parkinsonian action on male Wistar rats against rotenone induced Parkinsonian affect.^18^ A single case study of self‐medication of *Ganoderma lucidium* in which a 50‐year‐old male showed positive effects by improving motor skills, thought process, as well as emotion regulation, strengthens the clinical research findings.^19^ Thus, GAA can be used for in vitro and vivo studies, which can lead to drug development of PD.

## 5 CONCLUSION

*Ganoderma lucidum* exhibits cytotoxic, hepatoprotective, antioxidative, anticancer, and antinociceptive activities. This study predicted that *Ganoderma lucidum* could even be used to treat neurological disorders like PD. This study suggest that the best‐identified molecule against LRRK2 is GAA and it needs rigorous in vitro in vivo validations.

## AUTHOR CONTRIBUTIONS

Complete paper which includes idea generation, methodolgy, results, writing, editing and reviewing.

## FUNDING INFORMATION

Not received.

## CONFLICT OF INTEREST

The author proclaims no competing interests.
